# Expression of SSTR2a, FAP, HER2 and HER3 as potential radionuclide therapy targets in higher-grade meningioma

**DOI:** 10.1007/s00259-025-07075-8

**Published:** 2025-01-16

**Authors:** Maximilian J. Mair, Sabrina Hartenbach, Erwin Tomasich, Sybren L. N. Maas, Sarah A. Bosch, Georg Widhalm, Franziska Eckert, Felix Sahm, Johannes A. Hainfellner, Markus Hartenbach, Anna S. Berghoff, Matthias Preusser, Nathalie L. Albert

**Affiliations:** 1 Department of Nuclear Medicine, LMU Hospital, Ludwig Maximilians University Munich, Munich, Germany; 2 Division of Oncology, Department of Medicine I, Medical University of Vienna, Vienna, Austria; 3 MINUTEmedical GmbH, Vienna, Austria; 4 Department of Pathology, Leiden University Medical Center, Leiden, The Netherlands; 5 Department of Pathology, Erasmus MC Cancer Institute, University Medical Center Rotterdam, Rotterdam, The Netherlands; 6 Department of Neurosurgery, Medical University of Vienna, Vienna, Austria; 7 Department of Radiation Oncology, Medical University of Vienna, Vienna, Austria; 8 Department of Neuropathology, Institute of Pathology, Clinical Cooperation Unit Neuropathology, Ruprecht-Karls University Heidelberg, German Consortium for Translational Cancer Research (DKTK), German Cancer Research Center (DKFZ), Heidelberg, Germany; 9 Division of Neuropathology and Neurochemistry, Department of Neurology, Medical University of Vienna, Vienna, Austria; 10 Bavarian Cancer Research Center (BZKF), Partner Site Munich, Munich, Germany; 11 German Cancer Consortium (DKTK), Partner Site Munich, Munich, Germany; 12 Department of Nuclear Medicine, LMU Hospital, LMU Munich, Marchioninistraße 15, 81377 Munich, Germany

**Keywords:** Meningioma, Brain tumour, Theranostics, Somatostatin receptor, Fibroblast activating protein

## Abstract

**Purpose:**

High-grade meningiomas have high recurrence rates and limited prognosis. Radioligand therapies are approved in extracranial malignancies, but their value in brain tumours including meningiomas is unclear, as data on target expression is scarce.

**Methods:**

CNS WHO grade 2 and 3 meningioma samples were immunohistochemically stained for somatostatin receptor 2a (SSTR2a), fibroblast activation protein (FAP), and human epidermal growth factor receptors 2/3 (HER2/HER3). Target expression was correlated with (epi-)genetic tumour subtyping by DNA methylation analysis, genetic alterations, and survival.

**Results:**

Meningioma samples of 58 patients were included. SSTR2a expression (membranous/cytoplasmic) was observed in 43/55 (78.2%), and FAP expression in 15/58 (25.9%) evaluable samples, with HER2 and HER3 expression in one specimen each (1.7%). Membranous SSTR2a expression was strong in 18 (32.7%), intermediate in 12 (21.8%), and weak in 11 (20.0%) samples. While SSTR2a expression was more homogenous and mainly seen in regions with higher cellularity, FAP immunoreactivity was predominantly seen in tumour stroma and regions of lower cellularity. SSTR2a immunoreactivity was associated with *TRAF7* wildtype status (*p* = 0.034). FAP expression was more frequent in meningiomas of CNS WHO grade 3 (vs. CNS WHO 2; *p* < 0.001), and samples with *NF2* mutations (*p* = 0.032) or *CDKN2A/B* deletions (*p* = 0.013) compared to wildtype. FAP and SSTR2a expression (present vs. absent) were not associated with overall survival (*p* > 0.05).

**Conclusion:**

SSTR2a and FAP are expressed in high-grade meningioma samples to a variable extent, and differences across meningioma subtypes underscore the need for biomarkers to improve patient selection. Spatial heterogeneity of target expression should be considered in radioligand therapy design.

**Supplementary Information:**

The online version contains supplementary material available at 10.1007/s00259-025-07075-8.

## Introduction

Accounting for ~ 40% of intracranial tumours, meningiomas are the most commonly diagnosed primary neoplasms in the central nervous system (CNS) [1]. Due to their circumscribed and extra-axial localization, most meningiomas exhibit benign biological behaviour and are therefore frequently cured by sole resection [2]. However, about 20% of meningiomas show invasive growth patterns and/or atypical features and are therefore classified as either CNS WHO grade 2 or 3 based on histological and molecular characteristics such as *TERT* promoter mutations or homozygous deletions of *CDKN2A/B* [3]. In higher-grade meningiomas, further treatment modalities such as radiotherapy or stereotactic radiosurgery are needed depending on tumour grade and residual tumour volume. After exhaustion of local therapies, also systemic treatment can be applied albeit based on limited evidence [2]. In this regard, an increasing number of clinical trial initiatives investigating cytotoxic therapies, targeted agents, and immunotherapy underscores the unmet need in this patient population [4]. Indeed, overall survival in patients with high-grade meningioma and no further local therapeutic options reaches about 11 months in median [5]. In particular, systemic therapy may also be indicated in cases of extracranial metastasis to liver, lungs or bones, occurring rarely in higher grade meningioma and leading to severely impaired prognosis [6].

Combining the specificity of tumour-specific ligands with the cytotoxic activity of radionuclides, radioligand therapies have emerged as promising systemic treatment strategy and have been approved for use in prostate cancer ([^177^Lu]Lu-PSMA-617) as well as neuroendocrine tumours ([^177^Lu]Lu-DOTA-Tyr3-octreotate, [^177^Lu]Lu-DOTATATE) based on pivotal trial results [7, 8]. In the latter, the abundant expression of somatostatin receptor (SSTR) family members is harnessed to maximize antitumoral efficacy while minimizing systemic off-target effects [9]. Similarly, SSTR expression has been shown in meningiomas [10], and early phase trials of somatostatin receptor antagonists such as pasireotide or octreotide have been performed [11, 12]. Accordingly, SSTR-directed positron emission tomography (PET) using [^68^Ga]Ga-DOTATATE, [^68^Ga]Ga-DOTA-Tyr3-octreotide ([^68^Ga]Ga-DOTATOC), and [^68^Ga]Ga-DOTA-1-NaI(3)-octreotide ([^68^Ga]Ga-DOTANOC) is used in preoperative and pre-radiotherapy planning, to distinguish treatment-related changes from recurrence, and in challenging locations with unclear tumour extent [2]. With regard to therapeutic applications, data from case series and small prospective trials have shown therapeutic responses after radionuclide treatment with SSTR ligands linked to lutetium-177 or yttrium-90 [13, 14], but further investigation is warranted and ongoing.

However, responses towards SSTR-targeted radionuclide treatment and SSTR expression are variable. For instance, specific subgroups such as neurofibromatosis type 2-associated meningiomas show lower SSTR expression compared to others [15], underscoring the urgent need to systematically explore radioligand target expression across molecular subtypes [16]. Another emerging target is fibroblast activation protein (FAP), a transmembrane serine protease expressed on cancer-associated fibroblasts, tumour cells of various entities and also in fibrotic processes, but not in benign conditions or resting fibroblasts in adult tissue, including normal brain samples [17–19]. Based on these marked differences in FAP expression, diagnostic and therapeutic radionuclide approaches targeting FAP showed promising results in preclinical studies and small case series of various extracranial malignancies [20, 21]. Similarly, members of the human epidermal growth receptor family such as HER2 and HER3 have been investigated for theranostic approaches in extracranial tumours [22], while prior studies have shown conflicting results on target expression in meningioma [23–26].

Here, we evaluated the expression of SSTR2a, FAP, HER2 and HER3 in a molecularly characterized cohort of patients with CNS WHO grade 2 and 3 meningioma.

## Patients and methods

### Patient cohort

Adult patients (age ≥ 18 years) with meningiomas of CNS WHO grades 2 and 3 who were diagnosed at the Medical University of Vienna (Vienna, Austria) between 2000 and 2020 were identified from the Neuro-Biobank of the Medical University of Vienna and included in this retrospective study. Histological diagnosis was performed by a board-certified neuro-pathologist, and reclassification according to the WHO Classification of Central Nervous System Tumours 2021 was performed [3]. Accordingly, meningiomas with clear cell or chordoid histology, and/or 4–19 mitotic figures per 10 high-power fields (HPF, as approximation for 0.16 mm^2^ as defined by WHO 2021), and/or brain invasion and/or further atypical features were graded as CNS WHO grade 2. Meningiomas with *TERT* promoter mutation and/or *CDKN2A/B* homozygous deletion and/or ≥ 20 mitotic figures/10 HPF or carcinoma-, sarcoma-, or melanoma-like histology were considered as CNS WHO grade 3.

Data were collected retrospectively and stored in a FileMaker-based database (FileMaker Pro Advanced/Server 19, FileMaker Inc., Santa Clara, CA, USA) and handled in pseudonymized form. The study was approved by the ethics review board of the Medical University of Vienna (approval no. 2081/2022) and conducted according to institutional and national standards and compliant to the Helsinki Declaration of 1964 with all its amendments. Due to the retrospective nature of the study, obtainment of informed consent from included patients was waived.

### DNA methylation analysis and panel sequencing

DNA methylation profiling and methylation class allocation of these cases was available from previous analyses using Illumina EPIC 850k chips as described [27, 28]. Similarly, panel sequencing of tumour samples for *NF2*,* TRAF7*,* KLF4*,* SMO*,* AKT1*,* TERT* promotor, *ARID*,* SUFU* and *PIK3CA* for these cases had been performed for a previous investigation using the Illumina NextSeq 500 platform in paired-end mode [28].

### Immunohistochemistry

Sections of 4 μm were prepared from formalin-fixed, paraffin-embedded tumour samples. Immunohistochemical (IHC) stainings for SSTR2a and FAP were prepared using anti-SSTR2a rabbit monoclonal antibody (clone EP149, Cell Marque/Millipore Sigma, Rockin, CA, USA) and anti-FAP recombinant rabbit monoclonal antibody (clone JA56-11, Thermo Fisher Scientific/Invitrogen, Carlsbad, CA, USA). Stainings of HER2 and HER3 were performed using the Ventana Benchmark Ultra platform (Roche Diagnostics, Rotkreuz, Switzerland) using anti-HER2 rabbit monoclonal antibody (PATHWAY HER2 (clone 4B5), catalogue no. 790–4493, RRID: AB_2921204, ready-to-use [RTU], Roche) and anti-HER3 rabbit monoclonal antibody (clone D22C5, RRID: AB_2721919, 1:100, Cell Signaling Technology, Cambridge, UK) as described previously [29]. Positive controls and non-tumorous brain tissue samples were included (Supplementary Fig. 1). Here, pancreatic tissue samples (islet cells) were used as a positive control for SSTR2a and FAP, whereas breast cancer and lung cancer brain metastases were used for HER2 and HER3, respectively. Stainings of non-tumourous temporal lobe sections were negative for all stainings except SSTR2a as reported [30, 31].

Protein expression was evaluated semiquantitatively for SSTR2a (negative, 1+/2+/3+) and dichotomously for FAP given the overall lower staining intensity (positive vs. negative in the tumour stroma and on tumour cells; Supplementary Fig. 2), as well as HER2 and HER3. Spatial heterogeneity (areas with high/low cellularity; stroma; regions with angiogenetic activity) was described qualitatively. Haematoxylin/eosin slides for correlation were available from initial diagnostic workup.

### Statistical analysis

Results are given as absolute numbers and percentages, and independence of categorical variables (such as tumour subtypes with semiquantitative target expression) was assessed using Chi-square and Fisher’s exact test as appropriate. Distributions of metric variables between groups were compared using Mann-Whitney-U test. Overall survival (OS) was defined as the time between surgery and last follow-up or death, whereas progression-free survival (PFS) was defined as the time between surgery, last follow-up, or either progression/recurrence or death, and was illustrated using the Kaplan Meier method. Survival between groups was compared using the log-rank test. Further survival analysis in different subgroups such as WHO grades or according to genetic alterations was not feasible due to small sample size and a limited number of events. Statistical significance was defined as *p* ≤ 0.05, and analysis was conducted using R 4.4.1 using the packages *ggplot2*,* gridExtra*,* survival*,* survminer*, and *ComplexHeatmap* [32]. Given the exploratory and hypothesis-generating design of the study, no correction for multiple testing was performed [33].

## Results

### Baseline characteristics

Overall, 58 meningioma patients were included, of whom 35 (60.3%) were female. Median age was 60 years (range: 19–83). Most tumours were located at the cerebral convexity or falx/parasagittal (36/58, 62.1%), and 31/58 (53.4%) meningiomas were atypical according to initial histological diagnosis. Tumours were classified as CNS WHO grade 2 in 39/58 (67.2%), CNS WHO grade 3 in 18/58 (31.0%), and CNS WHO grade 1 in one tumour which was initially assigned WHO grade III based on previous classification due to rhabdoid histology but lacked further atypical or anaplastic features. Further baseline characteristics are given in Table 1.

**Table 1:** Baseline characteristics

	*n* = 58
**Sex**	
male	23 (39.7%)
female	35 (60.3%)
**Age at surgery** (median, range)	60 (19–83) years
**Tumour location**	
Cerebral convexity	21 (36.2%)
Falx/parasagittal	15 (25.9%)
Sphenoid wing	7 (12.1%)
Tentorium	5 (8.6%)
Posterior fossa	2 (1.7%)
*other (including cerebellopontine angle*,* intraorbital*,* petrous ridge*,* spinal)*	*4 (6.9%)*
*unknown*	*4 (6.9%)*
**Type of surgery**	
diagnosis/first surgery	39 (67.2%)
recurrence	13 (22.4%)
*unknown*	*6 (10.3%)*
**Documented radiotherapeutic treatment in clinical course**	
yes	22 (37.9%)
no	27 (63.8%)
*unknown*	*9 (15.5%)*
**Histology** (including original grading)	
atypical (WHO grade II)	30 (51.7%)
chordoid (WHO grade II)	9 (15.5%)
anaplastic (WHO grade III)	18 (31.0%)
rhabdoid (WHO grade III)	1 (1.7%)
**CNS WHO grade (WHO 2021)**	
CNS WHO grade 1	1 (1.7%)
CNS WHO grade 2	39 (67.2%)
CNS WHO grade 3	18 (31.0%)
**Meningioma methylation class**	
ben-1	10 (17.2%)
ben-2	9 (15.5%)
ben-3	4 (6.9%)
int-A	23 (39.7%)
int-B	4 (6.9%)
malignant	8 (13.8%)
**Prevalence of genetic alterations**	
*NF2* mutation	25 (43.1%)
*ARID1A/1B/2* mutation	13 (22.4%)
*TRAF7* mutation	8 (13.8%)
*CDKN2A/B* homozygous deletion	5 (8.6%)
*SMO* mutation	3 (5.2%)
*AKT1* mutation	2 (3.4%)
*SUFU* mutation	2 (3.4%)
*KLF4* mutation	1 (1.7%)
*TERT* promoter mutation	1 (1.7%)
**Progression-free survival (median**,** 95%CI)**	63.3 months (95%CI: 28.9– not reached)
**Overall survival (median**,** 95%CI)**	153 months (95%CI: 74.3– not reached)

### Expression of SSTR2a, FAP, HER2 and HER3 in meningioma samples

**Fig. 1:**
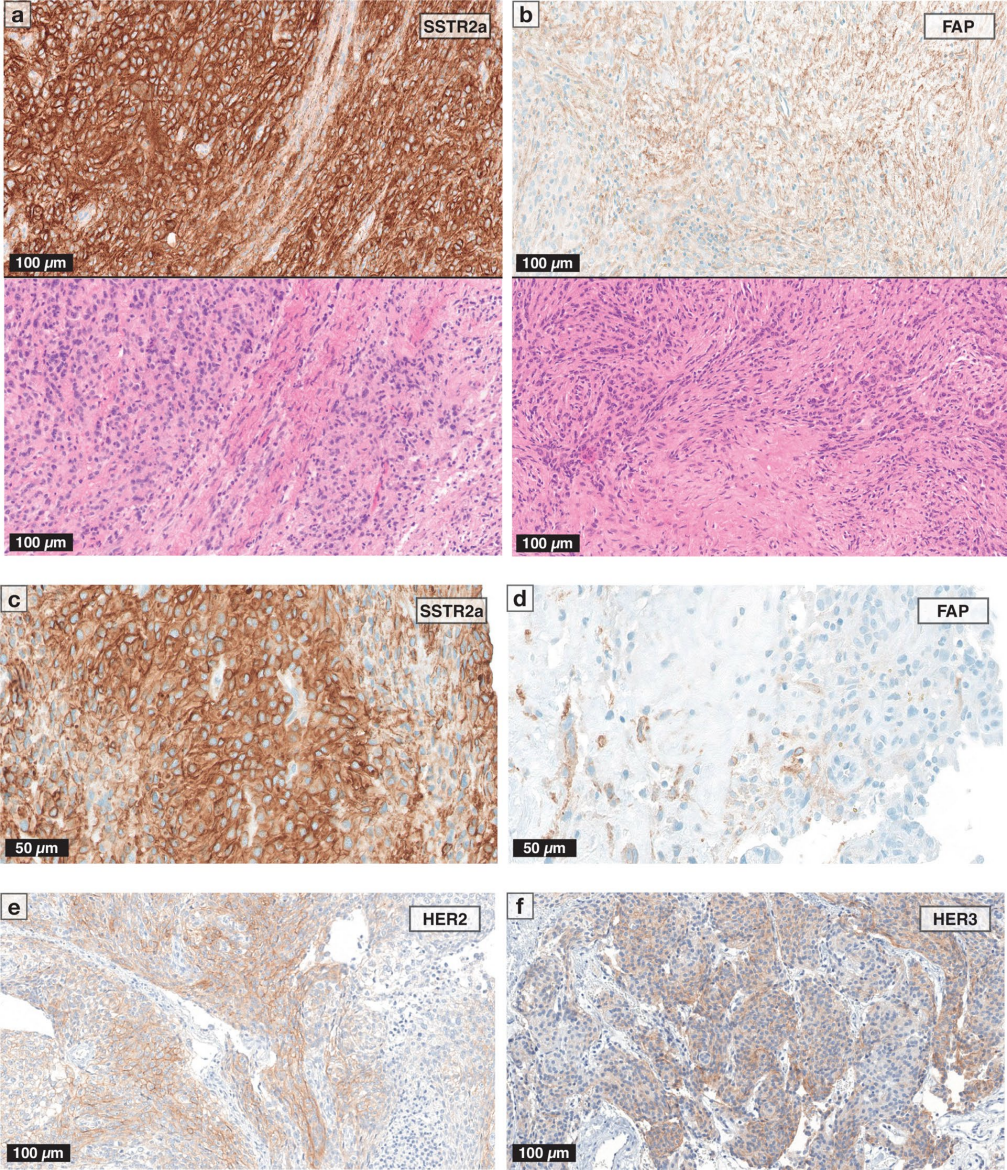
Immunohistochemical stainings of **SSTR2a (a/c)**, ** FAP (b/d)**, ** HER2 (e)**, ** HER3 (f)**. Magnification 200x (**a**, **b**, **e**, **f**) and 400x (**c**, **d**), scale bars (50 μm/100µm) as illustrated. Corresponding haematoxylin/eosin slides shown in lower panels of (**a**) and (**b**). FAP = fibroblast activation protein; HER2/HER3 = human epidermal growth receptor 2/3; SSTR2a = somatostatin receptor subtype 2a

Photographs of representative slides are given in Fig. 1. SSTR2a expression (either membranous, cytoplasmic, or both) was seen in 43/55 (78.2%) evaluable samples (Fig. 1a/c), while FAP expression was detected in 15/58 (25.9%) included samples (Fig. 1b/d). Membranous SSTR2a immunoreactivity was seen in 41/55 (74.5%) samples, of whom 18 (32.7%) showed strong (3+), 12 (21.8%) intermediate (2+), and 11 (20.0%) weak (1+) immunoreactivity. Cytoplasmic immunoreactivity was observed in 31/55 samples (56.4%), of whom intermediate cytoplasmic staining was seen in 20 (36.4%) and weak expression in 11 (20.0%) samples. In contrast, HER2 and HER3 expression was rarely observed, with only one sample each showing expression on tumour cells (Fig. 1e/f).

### Spatial heterogeneity and overlap of SSTR2a, FAP, HER2 and HER3 expression

In general, SSTR expression was homogenous and seen predominantly in regions with higher cellularity (Fig. 1a/c). FAP staining was seen in stroma-rich regions and areas of lower cellularity (Fig. 1b/d) and marked angiogenetic activity. Except for rare staining of vascular structures, no FAP immunoreactivity on non-tumoral structures was evident. Numerically, FAP expression was more frequent in SSTR2a-positive samples (14/43, 32.6%) than in those lacking immunoreactivity for SSTR2a (1/12, 8.3%; *p* = 0.147). The one HER2-positive sample showed also immunoreactivity for SSTR2a, whereas the HER3-positive sample displayed SSTR2a expression but lacked FAP immunoreactivity.

### Correlation of SSTR2a, FAP, HER2 and HER3 expression with clinical and molecular characteristics

**Fig. 2:**
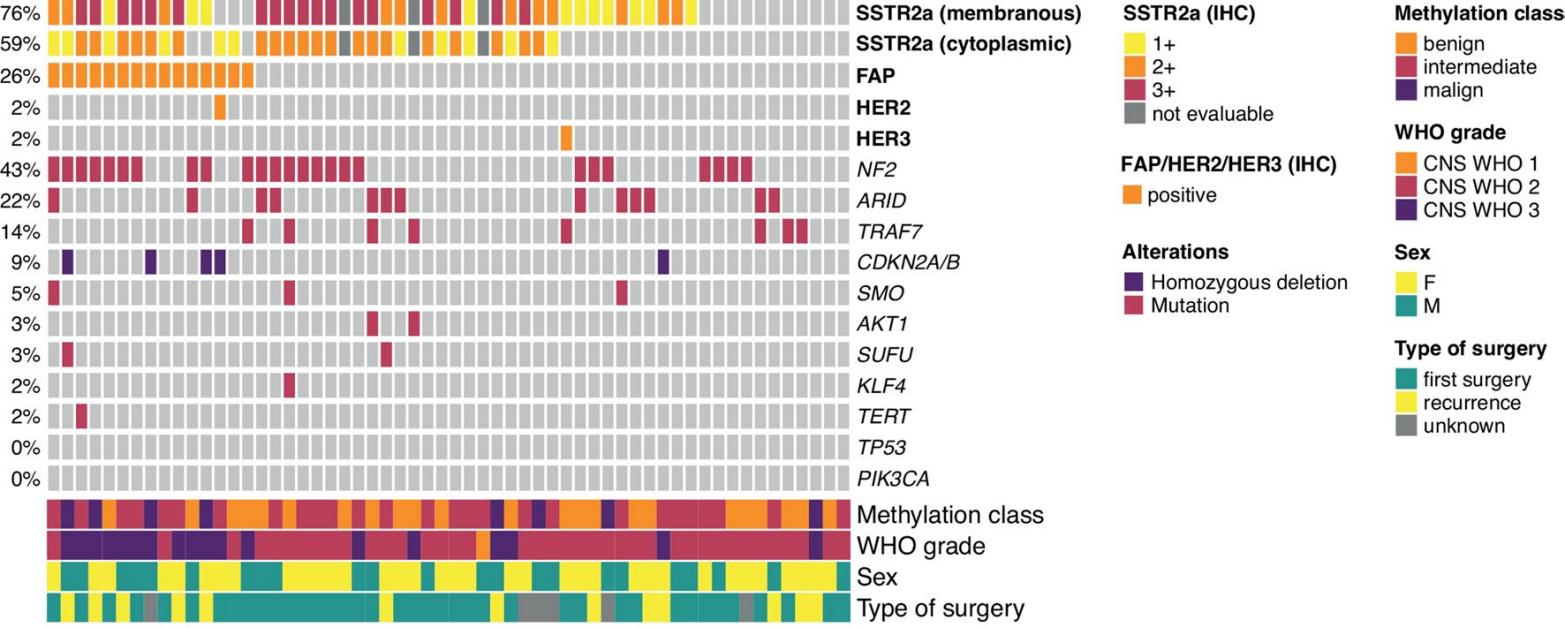
Associations between SSTR2a, FAP, HER2 and HER3 expression and clinical and molecular factors. FAP = fibroblast activation protein; HER2/HER3 = human epidermal growth receptor 2/3; IHC = immunohistochemistry; SSTR2a = somatostatin receptor subtype 2a

A summary of molecular alterations, methylation classes (MC) and target expression is given in Fig. 2.

**Fig. 3:**
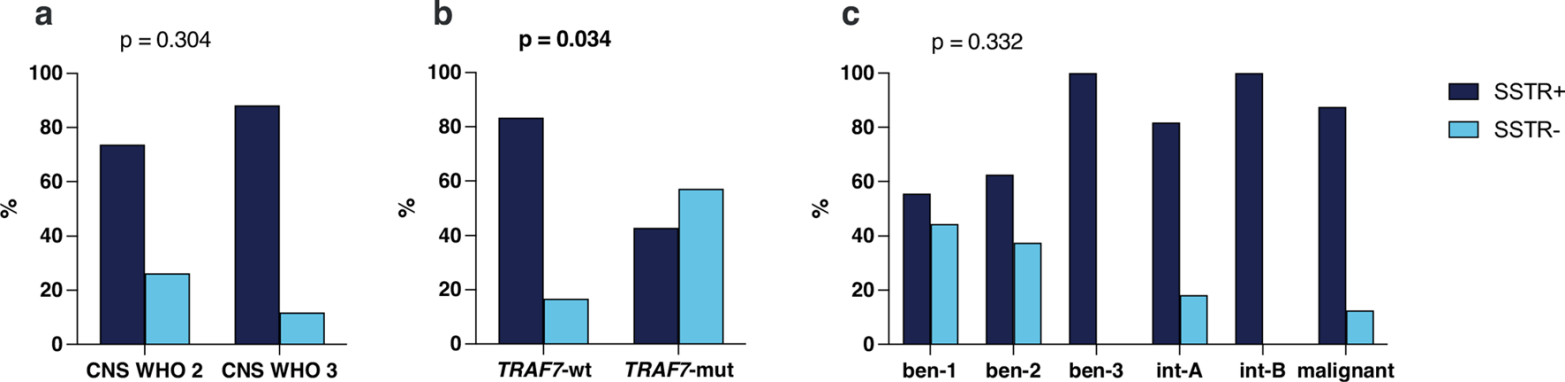
SSTR2a expression according to (**a**) CNS WHO grade, (**b**) TRAF7 mutational status, and (**c**) methylation class, ben 1/2/3 = methylation class benign 1/2/3; int-A/B = methylation class intermediate A/B; mut = mutant; wt = wildtype

SSTR2a expression (either membranous and/or cytoplasmic vs. no expression) did not correlate with CNS WHO grade, DNA methylation class or genetic alterations except *TRAF7* mutation, as 3/7 (42.9%) of *TRAF-*mutant showed SSTR2a immunoreactivity compared to 40/48 (83.3%) *TRAF-*wildtype samples (*p* = 0.034, Fig. 3a-c).

**Fig. 4:**
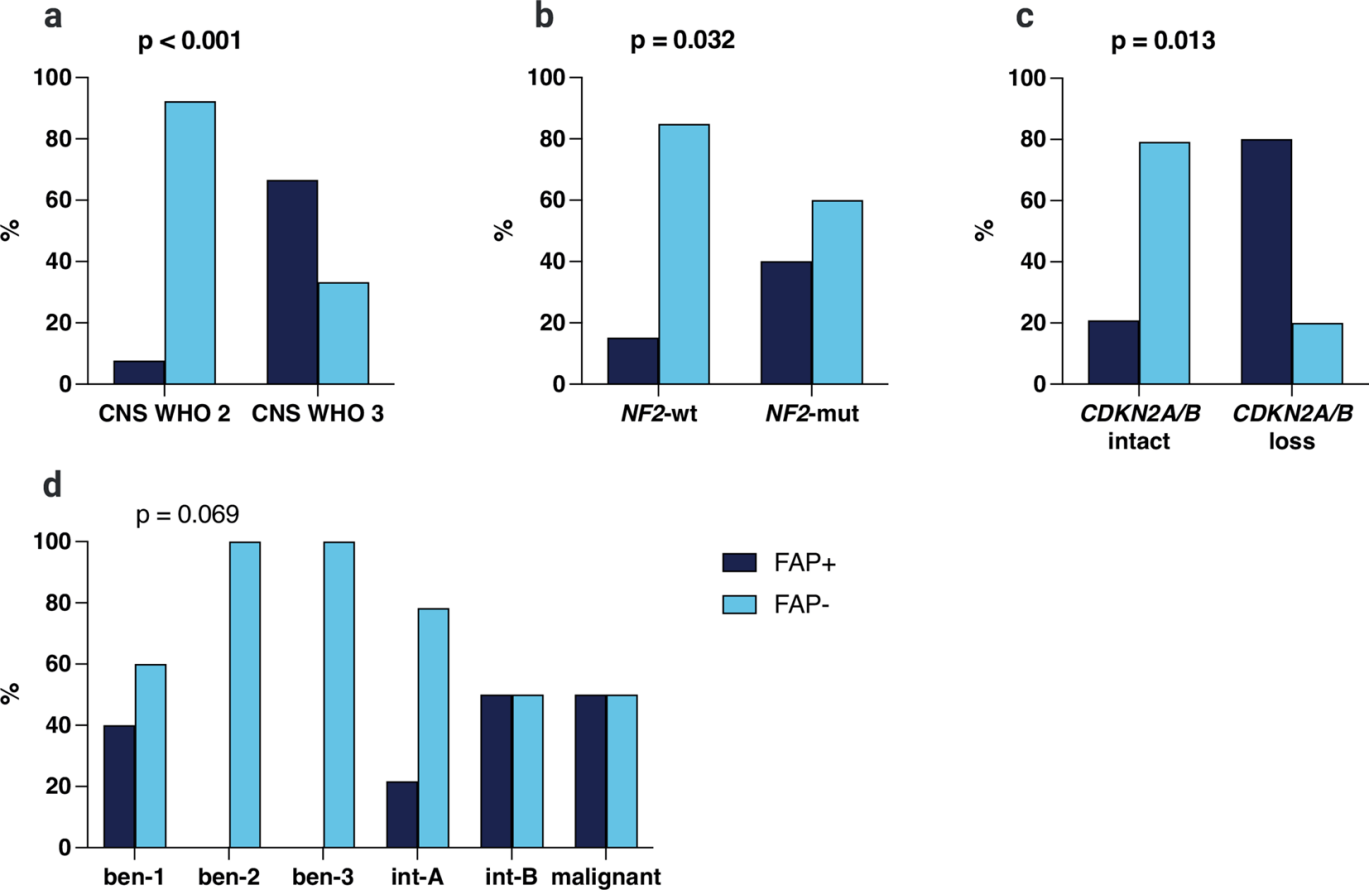
FAP expression according to (**a**) CNS WHO grade, (**b**) NF2 mutational status, (**c**) CDKN2A/B, and (**d**) methylation class. ben 1/2/3 = methylation class benign 1/2/3; int-A/B = methylation class intermediate A/B; mut = mutant; wt = wildtype

FAP expression was more frequently seen in CNS WHO grade 3 tumours (12/18, 66.7%) compared to CNS WHO grade 2 (3/39, 7.7%, *p* < 0.001, Fig. 4a). In terms of molecular alterations, FAP expression was more frequent in samples with *NF2* mutation (10/25, 40.0%) than in those with *NF2* wild-type (5/33, 15.2%, *p* = 0.032, Fig. 4b). Similarly, tumours with homozygous deletions of *CDKN2A/B* showed more often FAP expression (4/5, 80.0%) than their counterparts with intact *CDKN2A/B* (11/53, 20.8%, *p* = 0.013, Fig. 4c). No further correlations with molecular alterations including mutations of *ARID1A/ARID1B/ARID2*,* TRAF7*,* SMO*,* AKT1*,* SUFU*,* KLF4*, and the *TERT* promoter were observed. In addition, 4/8 (50.0%) samples of MC malignant and 2/4 (50.0%) of MC intermediate-B (int-B) showed FAP expression, followed by 4/10 (40.0%) with MC benign-1 (ben-1) and 5/23 (21.7%) with MC intermediate-A (int-A), whereas there were no FAP-positive samples in MC ben-2 and ben-3 (*p* = 0.069, Fig. 4d). Interestingly, patients with FAP-expressing meningiomas were slightly older than their FAP-negative counterparts (median: 63 [range: 54–76] vs. 58 years [19–83], *p* < 0.001), while there were no correlations with sex, tumour location, or type of surgery (first surgery/diagnosis vs. recurrence).

HER2 expression was observed in one meningioma sample of CNS WHO grade 3 of intraorbital location which also showed FAP expression and homozygous deletion of *CDKN2A/B.* Conversely, the HER3-positive sample was a CNS WHO grade 2 meningioma located in the posterior fossa with *TRAF7* mutation, but lacking FAP expression.

### Correlation of target expression with survival

**Fig. 5:**
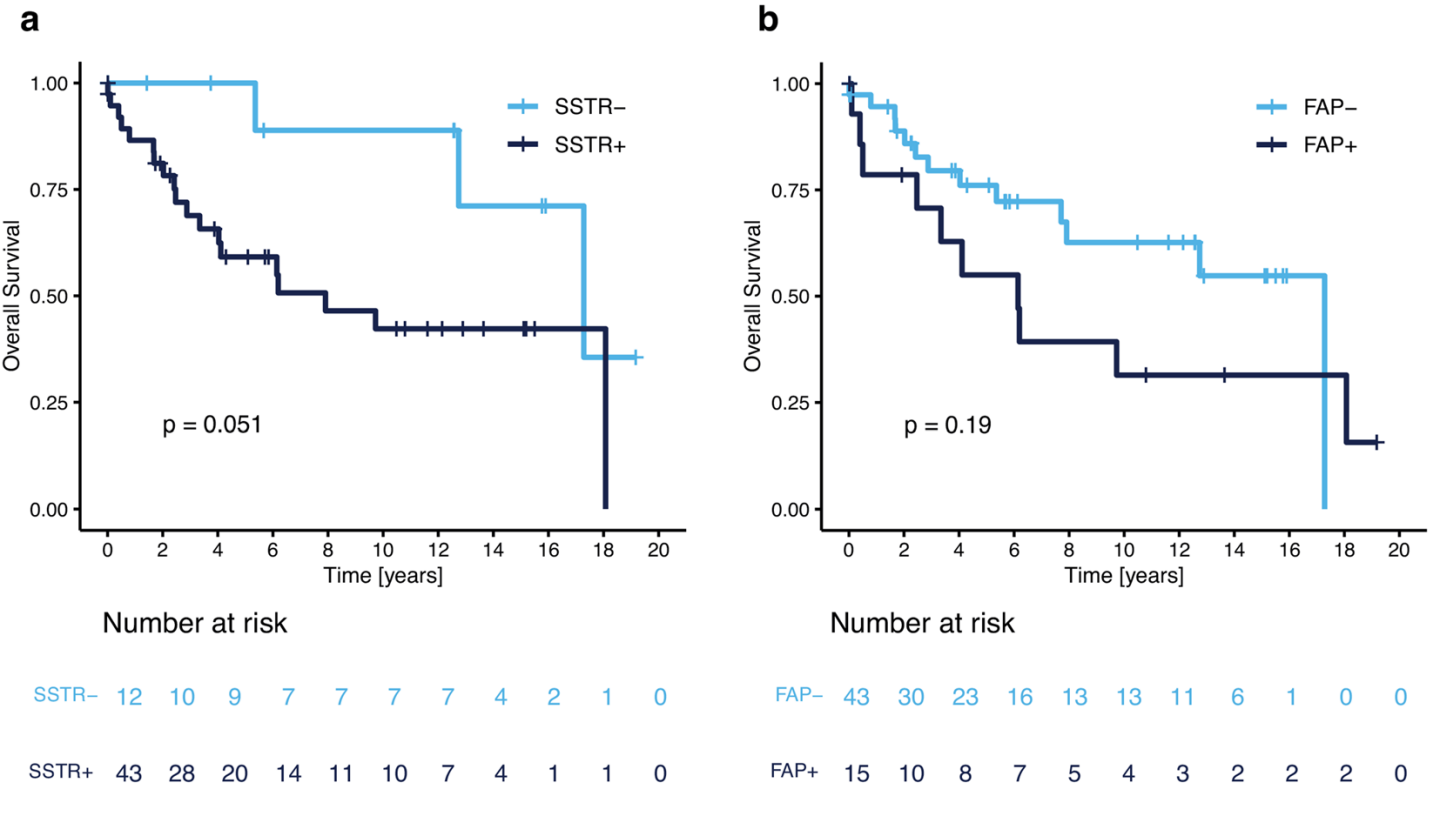
Overall survival according to (**a**) SSTR2a and (**b**) FAP expression. p-values as determined by log-rank test

Patients with samples showing no SSTR2a immunoreactivity had tendentially longer OS (median: 17.3 years [95%CI: 12.7– n.r.]) than their counterparts showing membranous and/or cytoplastic staining (median: 7.9 months [95%CI: 4.0– n.r.]; *p* = 0.051, Fig. 5a). In contrast, FAP expression did not correlate with OS (median [FAP-positive]: 6.1 years [95%CI: 3.3– not reached], vs. median [FAP-negative]: 17.3 years [95%CI: 7.9– not reached]; *p* = 0.19, Fig. 5b).

## Discussion

Herein, we evaluated the expression of several potential targets for radioligand treatment approaches in high-grade meningioma. SSTR2a and FAP were expressed in a subset of 78% and 26% of samples, respectively. In contrast, HER2/HER3 staining was only observed in one sample each. Previously, FAP expression had been evaluated in a wide array of extracranial tumours, with particularly high SUV_max_ on PET using ^68^Ga-linked FAP-targeting tracers in sarcomas and pancreatic carcinoma correlating with semiquantitative assessment of the number of FAP-positive cells in IHC staining of tumour tissue and stroma [34]. In line, early-stage clinical trials mainly included these entities and showed overall promising results when using a ^90^Y-based radioligand ([^90^Y]Y-FAPI-46) [35]. Also in meningioma, [^68^Ga]Ga-FAPI uptake was observed in case reports, particularly at tumour borders [36–38]. More recently, a case of a patient with rhabdoid meningioma receiving [^177^Lu]Lu-FAP-2286 treatment was reported, showing therapeutic responses of liver, bone, and pancreatic metastases after confirmation of FAP expression by pre-therapeutic [^68^Ga]Ga-FAP-2286 PET [39].

Still, a large-scale evaluation of SSTR2a and FAP expression in a molecularly characterized cohort of CNS WHO grade 2 and 3 meningioma samples was missing so far. Indeed, the presence of the target– either assessed by pretherapeutic PET scans or IHC in tumour tissue– is generally regarded as a prerequisite for a clinically relevant activity of radioligand therapies. For instance, a predictive value has been shown in patients with prostate cancer receiving [^177^Lu]Lu-PSMA-517 therapy [40] and patients with neuroendocrine tumours undergoing [^177^Lu]Lu-DOTATATE treatment [41]. Also in meningioma, SSTR2a expression correlated with progression-free survival in patients treated with [^177^Lu]Lu-DOTATATE according to a small retrospective analysis [42], although evidence for use of [^177^Lu]Lu-DOTATATE in meningeal neoplasms remains limited and further investigation is needed, ideally within prospective trials including investigations on predictive biomarkers [13]. This also extends to the still conflicting evidence on the correlation of SSTR2a expression with tumour grade and survival in meningioma [15, 43, 44].

In contrast to SSTR2a, FAP was only expressed in a subset of tumours. This highlights the importance of rational patient selection potentially benefitting from radionuclide treatments, particularly those targeting FAP. Importantly, FAP expression was numerically more frequent in SSTR2a-positive samples in our cohort. Moreover, we observed that FAP expression is particularly pronounced in tumours with *NF2*-mutant higher WHO grade or more malignant epigenetic subclass or tumours harbouring homozygous *CDKN2A/B* deletions. The classification of meningioma has undergone substantial changes in the last decade, and molecular testing has gained increasing importance in the prognostic assessment but also treatment decisions [3, 27, 45]. While our results remain exploratory given the small number of cases in certain subgroups, they underscore the need for pre-therapeutic evaluation of target expression.

While SSTR2a was expressed homogenously in regions of high cellularity, FAP-positive areas were predominantly found in stroma-rich regions and areas of lower cellularity, consistent with previous data in extracranial tumours [46]. Ideally, a theranostic target is expressed homogenously throughout the tumour and in all tumour manifestations to improve exposure to the treating radionuclide. However, the inter- and intralesional heterogeneity frequently hampers the efficacy of radioligand therapies [47]. In this regard, the choice of the emitting radionuclide remains of prime importance given the diverse ranges and linear energy transfer values of ɑ, β and Auger electron-emitting substances. Here, tandem approaches may represent a promising strategy to combine the higher range of β emitters with the improved linear energy transfer of ɑ-emitting radionuclides. Indeed, early data in prostate cancer alternating treatment of the β-emitting compound [^177^Lu]Lu-PSMA with the ɑ-emitting [^225^Ac]Ac-PSMA are encouraging [48, 49]. Moving forward, combined approaches aimed at distinct targets such as SSTR2a and FAP might further support overcoming treatment resistance and intratumoral heterogeneity.

Data on dosimetry are scarce in meningioma and underscore the high variability of absorbed doses upon [^177^Lu]Lu-DOTATATE treatment [50]. Radiation doses absorbed by the tumour are strongly dependent on exposure times and pharmacokinetic properties of the used radioligand. For FAP-targeted agents, a considerable number of different radioligands with varying retention times is under investigation in preclinical studies [21]. These include [^177^Lu]Lu-FAP-2286, [^177^Lu]Lu-FAPI-04, [^177^Lu]Lu-DOTA.SA.FAPI and [^177^Lu]Lu-DOTAGA.(SA.FAPi)_2_ as well as [^90^Y]Y-FAPI-46 using different chelators and radionuclides to improve intratumoral accumulation and to align the physical half-life with pharmacokinetic parameters. In the only case report on FAP-targeted radioligand treatment in metastatic rhabdoid meningioma, [^90^Y]Y-FAPI-46 has been used. However, dosimetry data are not reported, and further investigation is needed to identify the optimal compound [39].

This study has important limitations, mainly due to the retrospective design which is inherently linked to cohort heterogeneity and missing clinical data. In addition, small sample numbers in specific subgroups limit the generalisability of the results and preclude additional correlative analyses between target expression and (epi-)genetic classes as well as survival. Ideally, additional validation of SSTR and FAP expression using PET imaging would allow to assess target heterogeneity within patients, but was not feasible due to the retrospective nature of the study.

In conclusion, our data show that SSTR2a is expressed in most meningioma samples, and also FAP-targeted approaches hold therapeutic potential given the expression of FAP in a relevant subgroup. Whereas radioligand therapies targeting SSTR2a have shown promising activity in small, mainly retrospective studies, prospective randomised trials encompassing translational biomarker research are planned and in activation (such as LUMEN-1, NCT06326190). While FAP-targeting treatments are being investigated in early phase trials of extracranial tumours, our findings suggest that these agents might be more relevant as a complementary therapeutic approach (or as combination partners) in aggressive meningiomas, rather than as an alternative treatment option in tumours with absent or low SSTR2a expression.

## Electronic supplementary material

Below is the link to the electronic supplementary material.

Supplementary Material 1

## Data Availability

The datasets generated during and/or analysed during the current study are available from the corresponding author on reasonable request.
