# Supplementary material for: Expression of SSTR2a, FAP, HER2 and HER3 as potential radionuclide therapy targets in higher-grade meningioma
Source: Eur J Nucl Med Mol Imaging. 2025 Jan 16;52(8):2771–81. doi: 10.1007/s00259-025-07075-8 (PMC12162718; doi:10.1007/s00259-025-07075-8)
Supplement: Supplementary file 1 — Supplementary Material 1 [file 259_2025_7075_MOESM1_ESM.docx]

**Expression of SSTR2a, FAP, HER2 and HER3 as potential radionuclide therapy targets in higher-grade meningioma**

Maximilian J. Mair (1,2), Sabrina Hartenbach (3), Erwin Tomasich (2), Sybren L. N. Maas (4, 5), Sarah A. Bosch (2), Georg Widhalm (6), Franziska Eckert (7), Felix Sahm (8), Johannes A. Hainfellner (9), Markus Hartenbach (3), Anna S. Berghoff (2), Matthias Preusser (2), Nathalie L. Albert (1)

**Corresponding author:**

Prof. Nathalie Albert, MD

Department of Nuclear Medicine

LMU Hospital, LMU Munich

Marchioninistraße 15

81377 Munich (Germany)

Tel: +49 89 4400 74646

Email: [nathalie.albert@med.uni-muenchen.de](mailto:nathalie.albert@med.uni-muenchen.de)

**Supplementary Material**

**Supplementary Figure 1.** SSTR2a, FAP, HER2 and HER3 staining in positive controls (left; SSTR2a/FAP: pancreatic tissue with positive islet cells; HER2: HER2+ breast cancer brain metastasis; HER3: HER3+ lung cancer brain metastasis) and non-tumourous temporal lobe tissue.

**Supplementary Figure 2.** SSTR2a expression grading for stromal/cytoplasmic and membranous staining.
